# Changes in Objectively-Determined Walkability and Physical Activity in Adults: A Quasi-Longitudinal Residential Relocation Study

**DOI:** 10.3390/ijerph14050551

**Published:** 2017-05-22

**Authors:** Gavin R. McCormack, Lindsay McLaren, Grazia Salvo, Anita Blackstaffe

**Affiliations:** Department of Community Health Science, Cumming School of Medicine, University of Calgary, Calgary, AB T2N 1N4, Canada; lmclaren@ucalgary.ca (L.M.); grazia.salvo@ucalgary.ca (G.S.); ambortni@ucalgary.ca (A.B.)

**Keywords:** built environment, residential relocation, natural experiment, longitudinal, physical activity, walkability, walking, cycling, neighbourhood

## Abstract

Causal evidence for the built environment’s role in supporting physical activity is needed to inform land use and transportation policies. This quasi-longitudinal residential relocation study compared within-person changes in self-reported transportation walking, transportation cycling, and overall physical activity during the past 12 months among adults who did and did not move to a different neighbourhood. In 2014, a random sample of adults from 12 neighbourhoods (Calgary, AB, Canada) with varying urban form and socioeconomic status provided complete self-administered questionnaire data (*n* = 915). Participants, some of whom moved neighbourhood during the past 12 months (*n* = 95), reported their perceived change in transportation walking and cycling, and overall physical activity during that period. The questionnaire also captured residential self-selection, and sociodemographic and health characteristics. Walk Scores^®^ were linked to each participant’s current and previous neighbourhood and three groups identified: walkability “improvers” (*n* = 48); “decliners” (*n* = 47), and; “maintainers” (*n* = 820). Perceived change in physical activity was compared between the three groups using propensity score covariate-adjusted Firth logistic regression (odds ratios: OR). Compared with walkability maintainers, walkability decliners (OR 4.37) and improvers (OR 4.14) were more likely (*p* < 0.05) to report an increase in their transportation walking since moving neighbourhood, while walkability decliners were also more likely (OR 3.17) to report decreasing their transportation walking since moving. Walkability improvers were more likely than maintainers to increase their transportation cycling since moving neighbourhood (OR 4.22). Temporal changes in neighbourhood walkability resulting from residential relocation appear to be associated with reported temporal changes in transportation walking and cycling in adults.

## 1. Background

Urban and transportation planning policies should reflect the most rigorous public health evidence available [1]. Encouragingly, natural experiments have begun to emerge that can provide rigorous causal evidence for the influence of the built environment on physical activity [2,3,4,5]. These natural experiments can be broadly grouped into (1) “built environment intervention” studies that involve built environment modification with pre-and post-modification measurement of physical activity with either the same or different pre- and post-samples; or (2) “residential relocation” longitudinal studies where changes in individuals’ built environment and physical activity are measured before and after they relocate to a different neighbourhood [6]. This paper focuses on the latter.

Studies taking advantage of residential relocation have found support for associations between changes in the built environment and physical activity [7,8,9,10,11,12]. A Western Australian study (“RESIDE”) investigating longitudinal changes in physical activity and neighbourhood built characteristics before and after residential relocation to new homes (approximately 2–3 years later) found that increases in objectively-measured residential density, self-reported park access, and number of recreational destinations were associated with increased reported transportation cycling among urban adults [8]. Increases in objectively-measured street connectivity with relocation was also associated with increases in recreational cycling [8]. In a separate analysis [7] from the same study, increases in the number of objectively-measured transportation destinations and perceived transportation-related walkability characteristics were associated with an increase in duration of transportation walking; additionally, increases in the number of objectively-measured recreational destinations and perceived recreation-related walkability were associated with increases in duration of neighbourhood-based recreational walking. Other residential relocation longitudinal studies have also found changes in street connectivity [9], availability of recreational facilities [10], population density [11] and walkability (“Walk Score^®^”) [12] to be associated with changes in physical activity, among U.S. adults.

Compared with cross-sectional studies, longitudinal residential relocation studies can provide more precise estimates of the built environment–physical activity relationship, including accounting for residential self-selection (i.e., an individual choosing to reside in a neighbourhood with specific characteristics that support their current lifestyle or behavioural preferences). This study design however, requires repeated intra-individual data collections before and after residential relocation which is resource intensive and vulnerable to attrition [7]. “Quasi-longitudinal” residential relocation studies offer an alternative approach to investigating temporal relations between the built environment and physical activity. Compared with prospective longitudinal studies, estimates of change in physical activity from quasi-longitudinal studies are less precise; however, these estimates still provide relative measures of the magnitude and direction of physical activity change [13,14]. For instance, Aditjandra et al. [15] found that among British adults from ten neighbourhoods who had moved during the last eight years, increases in self-reported safety, travel accessibility, outdoor space availability, and neighbourhood attractiveness following relocation were associated with increases in self-reported transportation walking. Milakis et al. [16] found that decreases in self-reported access to public transport following relocation were associated with a decrease in self-reported walking, while lower perceived safety of cycling conditions was associated with lower bicycle use, among adults relocating from USA to Greek neighbourhoods. Among USA adults, Handy et al. [13] found positive associations between changes in self-reported physical activity opportunities, attractiveness, and safety and changes in reported neighbourhood physical activity following relocation. Cao et al. [17] modeled the neighbourhood choice process using structural equation modelling and found that positive changes in self-reported built characteristics were associated with self-reported changes in walking since relocating. In contrast to other quasi-longitudinal studies, Scheiner and Holz-Rau [18] found a decrease in self-reported frequency of transportation walking and cycling among those relocating to more suburbanized German municipalities.

A strength of quasi-longitudinal residential relocation studies so far has been their consistent adjustment for residential self-selection in estimates of association between the built environment and physical activity [13,15,16,17,18,19]. However, many quasi-longitudinal studies estimate change in the built environment based on self-report measures of the built environment [13,15,16,17,20]. Concordance between self-reported and objective-measures of the same built characteristics have been shown to be only low to moderate [21,22,23]. Furthermore, associations between self-reported physical activity and built environment characteristics may be inflated due to same source bias [19].

Quasi-longitudinal studies have measured within-person changes in physical activity behaviour within 1 year [13,17,20], 8 years [15], or 14 years [18] since neighbourhood relocation; and some studies have placed no restriction on lapsed time since relocation [16]. As the amount of lapsed time since relocation increases, the number of competing alternative explanations for the observed associations between the built environment and physical activity accumulates (especially in studies without an equivalent control group). The inclusion of a comparison (control) group allows the absolute magnitude of the change in physical activity in relation to the change in the built environment to be estimated—i.e., not just a cross-sectional estimate of association. Although non-movers have been included as a comparison group in some prospective longitudinal studies [10], they have typically been excluded from quasi-longitudinal studies [7,17,20,24] despite their value as a control group. One quasi-longitudinal study that included control groups examined transit and car use but not physical activity outcomes [19].

Building on previous evidence, the aim of this quasi-longitudinal study was to compare within-person changes in self-reported transportation walking, transportation cycling, and overall physical activity during the past 12 months between those who (1) relocated to a more walkable neighbourhood; (2) relocated to a less walkable neighbourhood, or; (3) did not relocate to a different neighbourhood or moved to a neighbourhood with the same walkability.

## 2. Method

### 2.1. Study and Sample Design

The study and sample design have been described in detail elsewhere [25]. Briefly, using stratified random sampling, 12 pre-1980 built Calgary neighbourhoods were selected as recruitment sites. The 12 neighbourhoods were randomly selected from strata defined by block pattern (grid, warped-grid, and curvilinear [26]) and socioeconomic status quartiles (“advantaged”, “somewhat advantaged”, “somewhat disadvantaged”, and “disadvantaged”) to represent the range in socioeconomic and urban form characteristics. As described elsewhere [25], we applied principal component analysis to Statistics Canada 2006 Census dissemination data that had been aggregated to the neighbourhood-level to derive our socioeconomic index. We used findings from Pampalon et al. [27] to inform our index which included seven census variables: the proportion of 25–64 year olds whose highest education is below a high school diploma; the proportion of single-parent families; the proportion of rented private dwellings; the proportion of divorced, separated, or widowed among those ≥15 years of age; the proportion unemployed among those ≥25 years of age; median gross household income; and average value of dwellings.

In April 2014, a simple random sample of households (*n* = 10,500) from the 12 neighbourhoods was mailed a survey package which included instructions for completing online versions of: (1) a physical activity, health and demographic questionnaire (PAHDQ); and (2) the Canadian Diet History Questionnaire II (C-DHQ II). The current paper presents data from the PAHDQ only. One participant per household ≥20 years of age was recruited. Of the 10,500 households sent the survey package, 407 were non-deliverable, and 918 completed the online PAHDQ. While not part of the recruitment protocol, at the request of a further 105 participants, we mailed-out a paper copy version of the PAHDQ. One week prior to sending the survey package to households, we sent a postcard introducing the “Pathways to Health” study. In addition, we sent two reminder postcards two and four weeks after sending the survey package to encourage participants to complete the survey. Participants also entered a prize draw for a US$50 voucher if they completed the survey. Despite our recruitment approach, the response rate, which does not consider access to the internet nor those who were ineligible to participate due to their age or language barriers, was only 10.1%. For the variables included in the current study, 915 participants provided complete data. Our sample included higher proportions of women, whites, those with post-secondary education, those married or common-law, and those without children <18 years at home and was older and had higher income compared with the 2014 Calgary civic census population data for the 12 study neighbourhoods.

### 2.2. Group Membership Based on Walkability

As part of the PAHDQ, participants reported whether they had relocated neighbourhood during the past 12 months. Movers reported the name or postal code of their previous and current residential neighbourhood. We used postal code to determine the participant’s neighbourhood, when a neighbourhood name was not provided. Using the Walk Score^®^ website (www.walkscore.com) we obtain the walkability score for each previous and current neighbourhood and linked these data to PAHDQ data for each study participant. We used Walk Score^®^ at the neighbourhood-level to decrease the loss of participants who did not report postal code for their previous neighbourhood. Walk Score^®^ includes straight-line distance estimates from households to nine commonly used amenities (grocery stores, restaurants, shopping, coffee shops, banks, parks, schools, bookstores, and entertainment). Using a decay function, distances to each amenity are weighted, and summed to create a walkability score (low = 0 to high = 100). Walk Score^®^ has been correlated with other multi-attribute walkability indices [28,29]. We only included participants who relocated from a Canadian residential neighbourhood. A total of *n* = 95 movers, which included 78 from within and 17 from outside the province, were categorized into those who relocated to a (1) more walkable neighbourhood (*n* = 48 walkability “improvers”) and (2) less walkable neighbourhood (*n* = 47 walkability “decliners”) based on their absolute within-person change in Walk Score^®^. The *n* = 14 participants who reported moving in the past 12 months, but had relocated from a non-Canadian neighbourhood or provided insufficient address information were grouped with the *n* = 806 participant who had not moved in the past 12 months. We assumed no change in the built environment during the past year among non-movers [20] thus these participants were assigned the Walk Score^®^ of their current neighbourhood (*n* = 820 walkability “maintainers”). Our decision to group those who moved from non-Canadian neighbourhoods with non-movers would tend to bias effects (e.g., differences between improvers/decliners and non-movers) towards the null. The inclusion of a control group in quasi-experimental and quasi-longitudinal studies is important from the perspective of estimating the counterfactual (the average change in physical activity in the absence of residential relocation) and for preserving internal validity.

### 2.3. Variables

#### 2.3.1. Physical Activity Change

For movers, three PAHDQ items each with five response options (increased a lot, increased somewhat, no change, decreased somewhat, and decreased a lot) captured participants’ perceived direction and relative magnitude of change in their transportation walking, transportation cycling, and overall physical activity since moving to their new neighbourhood within the past 12-month. Similarly, for non-movers, equivalently worded-items captured participants’ perceived change in transportation walking, transportation cycling, and overall physical activity in the past 12 months (increased a lot, increased somewhat, no change, decreased somewhat, and decreased a lot). Similar items, capturing perceived (quasi-longitudinal) changes in physical activity have been used elsewhere [15,20] and have acceptable test–retest reliability [13]. Responses to each item were collapsed into three categories (perceived “increase”, “no change”, or “decrease”). While these items may lack measurement precision, they maximize recall accuracy [14,17,18].

#### 2.3.2. Residential Self-Selection

To obtain independent estimates of the changes in neighbourhood walkability associated with perceived changes in the physical activity outcomes, it was necessary to collect and statistically adjust for residential self-selection. Thirteen items measured the importance (not at all, somewhat, and very important) of neighbourhood characteristics that informed participants’ choices to reside in their current neighbourhoods. The items captured the importance of proximity to transit, recreational and non-recreational destinations, work, schools, and downtown, access to highways and major roads and community associations, sense of community, attractiveness and cleanliness of streets, housing type variety, and quality of recreational facilities. These items have acceptable test–retest reliability and are associated with objectively-measured neighbourhood walkability [30]. Responses to each item were collapsed into “not important” and “important”.

#### 2.3.3. Sociodemographic Characteristics

The PAHDQ included sociodemographic items capturing participants’ sex, age, ethnicity (white or other), highest education attained (high school or less, college, or university), gross annual household income (<$60,000, $60,000 to 119,000, ≥$120,000, or do not know/refused to answer), marital status (married/common law or other), number of children at home <18 years of age (at least one or none), dog ownership in past 12 months (owner or non-owner), and motor vehicle access (always/sometimes or never/do not drive). Self-reported mental and physical health (poor, fair, good, very good, or excellent) and presence of an injury in the past 12 months that interrupted usual physical activity were also captured.

### 2.4. Analysis

#### 2.4.1. Descriptive Analysis of Walk Score^®^ and Participant Characteristics

ANOVA with Fisher’s Least Significance Difference (LSD) post hoc tests, was used to compare pre-move and post-move walkability scores (Walk Score^®^) between walkability “maintainers”, “improvers”, and “decliners”. Dependent t-tests were used to estimate the change in the Walk Score^®^ between pre-move and post-move among walkability “improvers” and “decliners”. Participant characteristics were compared between walkability “maintainers”, “improvers”, and “decliners” using ANOVA with LSD post hoc tests for continuous variables and Pearson’s Chi-square with post hoc z-tests of proportions for categorical variables.

#### 2.4.2. Propensity Score Estimation

We used propensity scores [31] to statistically adjust for differences in participant characteristics between walkability “improvers”, “decliners”, and “maintainers”. Propensity scores have been used elsewhere to adjust for covariate differences in estimated associations between neighbourhood walkability and physical activity [30]. Specifically, we compared the three groups using a multiple propensity score analysis [32] to estimate the conditional probabilities of a participant being a walkability “improver”, “decliner”, and “maintainer” given their observed residential self-selection, sociodemographic, and health-related characteristics. Propensity scores were estimated using multinomial logistic regression, where walkability group was regressed on all residential self-selection, sociodemographic, and health-related variables. Balancing observed characteristics of participants across the groups reduces confounding and bias in estimates of the group (or change in walkability) effect on perceived change in physical activity. To check that average participant characteristics were equivalent between the three groups after propensity score covariate-adjustment, we ran Analysis of Covariance (ANCOVA) (for continuous variables) and multinomial logistic regression (for categorical variables) models containing the estimated propensity scores. If there are no statistically significant differences in participant characteristics between the three walkability groups after propensity score adjustment, then the groups are considered balanced and therefore any observed differences in physical activity between the groups for physical activity is most likely the result of a change in neighbourhood walkability and not a result of observed confounding.

#### 2.4.3. Propensity Score Covariate-Adjusted Models

Firth Binary Logistic Regression odds ratios (OR) and 95 percent confidence intervals (95% CI) estimated the likelihood of (1) perceived increase versus no perceived change in physical activity and (2) perceived decrease versus no perceived change in physical activity for walkability improvers and walkability decliners relative to walkability maintainers (reference group). To adjust for covariates, in addition to change in walkability variable, two of the three estimated propensity scores were also entered as covariates (one propensity score is redundant, thus only two were included in the model). Adjusted models were estimated for each of the three outcome variables (i.e., perceived change in transportation walking, transportation cycling, and overall physical activity). We used Firth logistic regression (estimated using a penalized likelihood function) instead of traditional logistic regression (estimated using maximum likelihood) to reduce small-sample bias in our estimated odds ratios and standard errors. Firth logistic regression was undertaken using the R extension command (logistf) in SPSS version 22 (SPSS Inc., Chicago, IL, USA). We also undertook ANCOVA to compare the differences in perceived change in physical activity (i.e., transportation walking, transportation cycling, and overall physical activity) as a continuous outcome (ordinal) between walkability improvers, decliners, and maintainers adjusting for covariates (propensity scores). For significant ANCOVA, we assessed group pairwise differences using Fisher’s Least Significance tests. *p*-values less than 0.05 were statistically significant.

## 3. Results

### 3.1. Walk Score^®^ Differences between the Walkability Maintainer, Improver, and Decliner Groups

Pre-move mean Walk Score^®^ was significantly (*p* < 0.05) higher for the walkability decliner (72.53) versus the walkability improver (48.24) and maintainer (60.33) groups (Table 1). Post-move mean Walk Score^®^ was significantly (*p* < 0.05) higher in the walkability improver (64.48) versus the walkability decliner (56.34) and maintainer (60.33) groups; however, there was no difference in post-move mean Walk Score^®^ between walkability decliners and maintainers. For both walkability improvers and decliners, changes in Walk Score^®^ from pre-move to post-move were statistically significant (*p* < 0.05) (Table 1).

### 3.2. Participant Differences between the Walkability Maintainer, Improver, and Decliner Groups

Relative to non-movers (walkability maintainers), those moving to a less walkable neighbourhood (walkability decliners) were significantly (*p* < 0.05) younger, included a higher proportion of non-white individuals, higher proportion currently residing in a socioeconomically advantaged neighbourhood, and lower proportion reporting proximity to schools as an important reason for choosing their current neighbourhood (Table 2). Notably, while not reaching statistical significance, a lower proportion of walkability decliners relative to maintainers and improvers reported proximity to transport as an important reason for moving to their current neighbourhood. Relative to non-movers, those moving to a more walkable neighbourhood (walkability improvers) were significantly (*p* < 0.05) younger, included a higher proportion of non-white individuals, higher proportion of dog owners, higher proportion reporting no injury in the past 12 months, and higher proportion residing in socioeconomically advantaged neighbourhoods.

### 3.3. Propensity Score Estimation and Covariate-Balance Check

Group membership (walkability maintainer, decliner, and improver) was regressed on all residential self-selection, sociodemographic, and health related variables to estimate the propensity scores. Based on ANCOVA and multinomial logistic regression results, we found no significant differences in residential self-selection, sociodemographic, and health-related variables among the three walkability groups after propensity score adjustment, suggesting that the three groups were statistically balanced regarding their observed covariates.

### 3.4. Perceived Change in Physical Activity by Walkability Maintainer, Improver, and Decliner Groups

Compared with 12% of walkability maintainers, approximately 40% of participants who relocated to a more walkable or to a less walkable neighbourhood perceived that their transportation walking had increased (Table 3). Adjusting for covariates, walkability maintainers, decliners (OR 4.37; 95% CI 1.98, 9.44) and improvers (OR 4.14; 95% CI 2.00, 8.43) were both more likely (*p* < 0.05) to perceive that their transportation walking had increased since relocating neighbourhood. Similar proportions of walkability maintainers and walkability improvers perceived a decrease in their transportation walking (14% and 16.1%, respectively) compared with 36.4% of walkability decliners who reported a perceived decrease in their transportation walking (Table 3). Adjusting for covariates, compared with maintainers, decliners were also more likely (*p* < 0.05) to decrease their transportation walking since moving (OR 3.17; 95% CI 1.43, 6.81).

Higher proportions of walkability decliners (17.1%) and improvers (21.4%) versus walkability maintainers (6.3%), perceived their transportation cycling had increased (Table 3). However, adjusting for covariates, compared with walkability maintainers, walkability improvers only were more likely (*p* < 0.05) to increase their transportation cycling since relocating neighbourhood (OR 4.22; 95% CI 1.65, 9.99) (Table 3). No statistically significant associations were found between walkability group and perceived change in overall physical activity.

Adjusting for covariates, we found higher perceived change in transportation walking (based on the continuous ordinal scale) for walkability improvers, compared with walkability maintainers and decliners (*p* < 0.05) (Table 4). There were no significant differences in perceived change in transportation cycling or overall physical activity continuous outcomes between the walkability maintainer, decliner, or improver groups.

## 4. Discussion

Our findings provide evidence of a temporal relationship between objectively-measured walkability and transportation walking. Findings from other quasi-longitudinal [13,15,16,17,20] and longitudinal [7,8] studies suggest that some participants may increase or decrease their physical activity after relocation. We also found that participants who relocated to either more and less walkable neighbourhoods in the past 12 months were both more and less likely to increase their transportation walking compared with non-movers. Notably, we found that only those participants who relocated to a less walkable neighbourhood were more likely to decrease their transportation walking in the past 12 months compared with non-movers. This suggests that moving to a more walkable neighbourhood is likely to result in either increased or similar levels of transportation walking following relocation, while those who move to a less walkable neighbourhood are at risk of their level of transportation walking decreasing following relocation. Similar to other longitudinal studies [8], we found that compared with non-movers, participants who moved to more walkable neighbourhoods were more likely to report increased transportation cycling. However, compared with non-movers, participants who relocated to a more or less walkable neighbourhood had a similar likelihood of increasing or decreasing overall physical activity. Our findings suggest that relocating to a more walkable neighbourhood may be beneficial in terms of increasing transportation walking.

Our findings support the specificity between the built characteristics and physical activity [30,33]. Like previous cross-sectional evidence, we found that the neighbourhood built environment was more strongly related to transportation walking and cycling compared with overall physical activity (which includes leisure and recreational activities) [34,35]. Not surprisingly, others have also found Walk Score^®^ to be positively associated with transportation walking [36,37]. A longitudinal study using Street Smart Walk Score^®^ found a positive association between walkability and changes in transportation walking and body mass index following relocation [12]. Walk Score^®^ captures the availability of primarily non-recreational destinations (i.e., stores, transit, and services) which likely explains our finding that changes in transportation walking and cycling were associated with changes in walkability because of relocation. Thus, using Walk Score^®^ might infer that we can draw stronger conclusions about the overall proximity and availability of destinations and their impact on change in transportation walking and cycling. Undertaking quasi-longitudinal analysis with other operational definitions of walkability, such as those used in previous longitudinal studies [7,8,9,10,11], could provide useful information about which built characteristics cause changes in walking, cycling, and other physical activities.

Handy et al. [13] found that changes in self-reported attractiveness, physical activity opportunities, and safety were associated with changes in neighbourhood physical activity. We however, found no relation between walkability and overall physical activity. This difference in findings is not surprising given that we used an objective-measure of walkability that may or may not have indirectly captured other neighbourhood characteristics such as attractiveness, physical activity opportunities and safety. We also found that participants did not decrease their overall physical activity following relocation to either a more or less walkable neighbourhood suggesting that the built environment might not be as important for supporting recreational physical activity. Alternatively, there may be a lag in exposure to a new neighbourhood built environment (i.e., longer than the 12 months) and its effect on physical activity [38]. Speculatively, this lag may also explain why some participants who relocated to a less walkable neighbourhood were more likely to increase their transportation walking, although it is also possible that these people are more inclined to explore their new surroundings regardless of the neighbourhood’s walkability. Residents within the first few months of arriving to a new neighbourhood may undergo a “honeymoon phase” whereby residents are highly satisfied with their new surroundings and they suppress or ignore previous reasons or preferences informing their decisions to relocate to the neighbourhood [39]. During this time, other incoming residents may undergo cognitive dissonance whereby expectations and preferences informing their decisions to relocate are not met or do not match the new neighbourhood environment surroundings. This arrival phase is typically followed by an adaptation phase whereby new residents, after some time, begin to adapt to their new neighbourhood surroundings [39]. Our findings where participants who moved to a less walkable neighbourhood perceived their transportation walking and cycling had increased, likely reflect the participant’s arrival phase response to their new environment. Studies designed to examine the moderating effect of exposure or time residing in the neighbourhood on the built environment–physical activity relationship are needed. Our findings highlight that moving to a less or more walkable neighbourhood will not have the same effect on physical activity for all individuals and therefore other interventions might also be required to promote physical activity post relocation.

The low response rate limits the generalizability of our findings. Our self-report measure of physical activity could be impacted by recall bias [17]. Due to limits on the available pre-and post-move data, our models adjusted for covariates but assume that these covariates are time-invariant. We statistically adjusted for observed differences, including residential self-selection, between walkability maintainers, decliners and improvers using propensity scores however, unmeasured confounders associated with group membership or neighbourhood choice and physical activity may still exist. Due to our small sample of movers, we were not able to use and test different Walk Score^®^ thresholds or cut points to define the walkability decliners and improvers. Future studies should investigate the practical and clinical relevance of different Walk Score^®^ or walkability thresholds in relation to physical activity outcomes. Our study included only a single objective measure of the built environment that focused primarily on estimating access to a small number of different types of destination. While studies have found moderate to strong correlations between Walk Score^®^ and other built characteristics supportive of physical activity [28,29], it is possible that neighbourhoods with low land use mix may still be highly walkable.

## 5. Conclusions

Our quasi-longitudinal findings provide evidence for a temporal relation, and are consistent with a causal relation, between walkability, as estimated by Walk Score^®^, and transportation walking and cycling. Our findings also suggest that walkability might result in a change in physical activity but that moving to a more walkable neighbourhood does not guarantee that the new residents will initially increase their transportation walking or cycling, or physical activity. Qualitative and quantitative studies are needed to identify other social and environmental factors that may explain “how” and “why” physical activity does or does not change following relocation to a new neighbourhood, which could inform policy for urban and transportation planning and public health.

## Figures and Tables

**Table 1:** Within and between residential relocation group differences in neighbourhood walkability.

Walkability (Walk Score^®^)	Residential Relocation Group		
	Non-Movers “Maintainers” (*n* = 820)	Moved to Less Walkable Neighbourhood “Decliners” (*n* = 47)	Moved to More Walkable Neighbourhood “Improvers” (*n* = 48)
	Mean ± SD	Mean ± SD	Mean ± SD
Pre-move (previous neighbourhood)	60.33 ± 12.30 ^a,b^	72.53 ± 15.40 ^a,c,Ψ^	48.24 ± 17.72 ^b,c,†^
Post-move (current neighbourhood)	60.33 ± 12.30 ^e,f^	56.34 ± 10.26 ^e,g,Ψ^	64.48 ± 11.98 ^f,g,†^
Current minus previous neighbourhood Walk Score^®^	0	−16.19 ± 11.36	16.24 ± 13.09

Study location: Calgary, Alberta, Canada. Non-movers include participants who did not report residential relocation during the past 12 months therefore pre-move and post-move Walk Score^®^ are the same. Between group differences in walkability for each pre-move and post-move tested using One-Way ANOVA with Fisher’s Least Significance pairwise post hoc comparisons. Estimates with the same letter superscript (^a, b, c, d, e, f, g^) are significantly different between groups at *p* < 0.05. Pre-move and post-move differences in walkability within each group tested using dependent t-tests. Estimates with the same symbol superscript (^Ψ, †^) are significantly different within groups at *p* < 0.05. SD: Standard Deviation.

**Table 2:** Sociodemographic characteristics and reasons for moving to current neighbourhood by residential relocation group (non-movers, moved to a more walkable neighbourhood, moved to a less walkable neighbourhood).

Sociodemographic Characteristics and Reasons for Moving to Current Neighbourhood		Residential Relocation Group		
		Non-Movers “Maintainers” (*n* = 820)	Moved to Less Walkable Neighbourhood “Decliners” (*n* = 47)	Moved to More Walkable Neighbourhood “Improvers” (*n* = 48)
Sociodemographic Characteristics		Estimate ^a^	Estimate ^a^	Estimate ^a^
Age in years *		54.4 ± 13.8	41.6 ± 16.7	42.8 ± 15.6
No. children <18 years		0.5 ± 0.9	0.3 ± 0.6	0.6 ± 0.9
Sex	Women	61.3	74.5	72.9
Ethnicity *	Non-white	10.5	14.9	25.0
Highest education achieved	High school	7.9	14.9	10.4
	College	19.9	17.0	18.8
	University	72.2	68.1	70.8
Gross household income	<$60,000/year	10.2	19.1	16.7
	$60,000–119,000/year	29.8	25.5	31.3
	≥120,000/year	43.2	42.6	41.7
	Do not know/refused	16.8	12.8	10.4
Marital status	Married/common-law	77.7	70.2	75.0
Dog ownership in past year *	Owner	30.9	38.3	54.2
Motor vehicle access	Never/do not drive	15.5	12.8	6.3
Injury in past year *	No injury	60.9	63.8	79.2
Current neighbourhood SES *	Most disadvantaged	40.1	36.2	18.8
	Somewhat disadvantaged	26.8	27.7	29.2
	Somewhat advantaged	19.9	14.9	22.9
	Most advantaged	13.2	21.3	29.2
**Reasons for neighbourhood choice**				
Proximity: transport	Important	60.0	44.7	62.5
Proximity: stores/services	Important	79.5	78.7	83.3
Proximity: rec. facilities	Important	83.2	80.9	75.0
Proximity: downtown	Important	76.5	72.3	83.3
Proximity to work	Important	77.7	70.2	83.3
Proximity to schools *	Important	67.3	42.6	54.2
Access: highways/major roads	Important	74.9	76.6	83.3
Access: community association	Important	41.5	29.8	39.6
Sense of community	Important	73.8	78.7	79.2
Attractiveness of streets	Important	82.3	74.5	70.8
Cleanliness of streets	Important	80.0	74.5	77.1
Variety of housing types	Important	64.6	55.3	56.3
Quality of recreation facilities	Important	81.2	76.6	79.2

Study location: Calgary, Alberta, Canada (2014); ^a^ Percent estimated for categorical variables and mean (±standard deviation) estimated for continuous variables. Pearson’s chi-square with z-score pairwise post hoc comparisons used for estimated differences in categorical variables and One-Way ANOVA with Fisher’s Least Significance Difference pairwise post hoc comparisons used for estimated differences in continuous variables. * *p* < 0.05.

**Table 3:** Firth binary logistic regression odds ratios (OR) and 95% confidence intervals (95% CI) for estimated associations between residential relocation/walkability group and participant’s perceived change in physical activity during the past 12-months.

Perceived Physical Activity Change in Past 12 Months		Residential Relocation by Walkability Group					
	*n*	Non-Movers ^a^ “Maintainers”		Moved to Less Walkable Neighbourhood “Decliners”		Moved to More Walkable Neighbourhood “Improvers”	
		% (*n*) Increased/Decreased PA	OR (Reference)	% (*n*) Increased/Decreased PA	OR (95% CI)	% (*n*) Increased/Decreased PA	OR (95% CI)
Walking for transportation (increase vs. no change)	796	12.4 (89)	1.00	40.0 (14)	4.37 (1.98, 9.44) *	39.5 (17)	4.14 (2.00, 8.43) *
Walking for transportation (decrease vs. no change)	795	14.0 (102)	1.00	36.4 (12)	3.17 (1.43, 6.81) *	16.1 (5)	1.17 (0.39, 2.97)
Cycling for transportation (increase vs. no change)	770	6.3 (44)	1.00	17.1 (12)	2.39 (0.78, 6.46)	21.4 (9)	4.22 (1.65, 9.99) *
Cycling for transportation (decrease vs. no change)	856	16.4 (127)	1.00	29.3 (12)	1.86 (0.86, 3.82)	15.4 (6)	1.02 (0.38, 2.39)
Overall physical activity (increase vs. no change)	745	33.7 (224)	1.00	50 (20)	1.57 (0.79, 3.12)	47.5 (19)	1.50 (0.75, 2.97)
Overall physical activity (decrease vs. no change)	652	26.0 (155)	1.00	25.9 (7)	1.04 (0.41, 2.35)	22.6 (8)	1.12 (0.47, 245)

Significantly different from reference group; * *p* < 0.05.

**Table 4:** Differences in participants’ perceived change in physical activity during the past 12 months by residential relocation/walkability group.

Perceived Physical Activity Change in Past 12 Months	Residential Relocation Group		
	Non-Movers “Maintainers” (*n* = 820)	Moved to Less Walkable Neighbourhood “Decliners” (*n* = 47)	Moved to More Walkable Neighbourhood “Improvers” (*n* = 48)
	Marginal Mean (95% CI) *	Marginal Mean (95% CI) *	Marginal Mean (95% CI) *
Walking for transportation	2.95 (2.90,3.00) ^a^	3.00 (2.78,3.22) ^b^	3.30 (3.08, 3.53) ^a,b^
Cycling for transportation	2.80 (2.75, 2.85)	2.74 (2.52, 2.97)	3.04 (2.85, 3.67)
Overall physical activity	3.11 (3.04, 3.17)	3.30 (3.02, 3.58)	3.24 (2.96, 3.52)

Study location: Calgary, Alberta, Canada. Perceived change in walking for transportation, walking for cycling, and overall physical activity measured on a 5-point ordinal scale (1 = decreased a lot, 2 = decreased somewhat, 3 = no change, 4 = increased somewhat, 5 = increased a lot). Non-movers include participants who did not report residential relocation during the past 12 months. * Estimated marginal mean adjusting for all covariates via propensity scores. Between group differences in physical activity compared using ANCOVA with Fisher’s Least Significance pairwise post hoc comparisons. Estimates with the same letter superscript (^a,b^) are significantly different between groups at *p* < 0.05.
